# Central Sleep Apnoea Is Related to the Severity and Short-Term Prognosis of Acute Coronary Syndrome

**DOI:** 10.1371/journal.pone.0167031

**Published:** 2016-11-23

**Authors:** Marina Florés, Jordi de Batlle, Alicia Sánchez-de-la-Torre, Manuel Sánchez-de-la-Torre, Albina Aldomá, Fernando Worner, Estefanía Galera, Asunción Seminario, Gerard Torres, Mireia Dalmases, Josep M. Montserrat, Onintza Garmendia, Ferran Barbé, on behalf of the Spanish Sleep Group

**Affiliations:** 1 Group of Translational Research in Respiratory Medicine, Hospital Universitari Arnau de Vilanova and Santa Maria, IRBLleida, Lleida, Spain; 2 Centro de Investigación Biomédica en Red de Enfermedades Respiratorias (CIBERES), Madrid, Spain; 3 Cardiology Department, Hospital Universitari Arnau de Vilanova, IRBLleida, Lleida, Spain; 4 Servicio de Neumología, Unidad del Sueño, Hospital Clínic de Barcelona, Universidad de Barcelona, Barcelona, Spain; University of Rome Tor Vergata, ITALY

## Abstract

**Objective:**

To evaluate the relation of central sleep apnoea (CSA) to the severity and short-term prognosis of patients who experience acute coronary syndrome (ACS).

**Methods:**

Observational study with cross-sectional and longitudinal analyses. Patients acutely admitted to participating hospitals because of ACS underwent respiratory polygraphy during the first 24 to 72 h. CSA was defined as an apnoea-hypopnoea index (AHI) >15 events•h^-1^ (>50% of central apnoeas). ACS severity (Killip class, ejection fraction, number of diseased vessels and peak plasma troponin) was evaluated at baseline, and short-term prognosis (length of hospitalization, complications and mortality) was evaluated at discharge.

**Results:**

A total of 68 CSA patients (AHI 31±18 events•h^−1^, 64±12 years, 87% males) and 92 controls (AHI 7±5 events•h^−1^, 62±12 years, 84% males) were included in the analyses. After adjusting for age, body mass index, hypertension and smoking status, patients diagnosed with CSA spent more days in the coronary unit compared with controls (3.7±2.9 vs. 1.5±1.7; p<0.001) and had a worse Killip class (Killip I: 16% vs. 96%; p<0.001). No differences were observed in ejection fraction estimates.

**Conclusions:**

CSA patients exhibited increased ACS severity as indicated by their Killip classification. These patients had a worse prognosis, with longer lengths of stay in the coronary care units. Our results highlight the relevance of CSA in patients suffering ACS episodes and suggest that diagnosing CSA may be a useful strategy to improve the management of certain ACS patients.

## Introduction

Cardiovascular diseases are the main cause of death worldwide, with 17.5 million victims reported in 2012 [1]. Coronary artery disease (CAD) is responsible for greater than half of all deaths from a cardiovascular cause. Acute coronary syndrome (ACS), which ranges from unstable angina to myocardial infarction, is often the first manifestation of a subject’s CAD [1, 2]. Central sleep apnoea (CSA) is a sleep disorder characterized by a temporary failure in centrally controlled breathing that results in a lack of drive to breathe during sleep. The prevalence of CSA is low in the general population and constitutes less than 5% of patients who have been referred to a sleep clinic [3]. However, the prevalence of CSA in cardiovascular patients is high, reaching up to 33 to 40% in heart failure (HF) patients [4]. Currently, the prevalence of CSA in ACS patients is unknown.

Among the different sleep disorders, CSA is considered to be the primary diagnosis when central apnoea is responsible for ≥50% of the total apnoea episodes [5]. In contrast, obstructive sleep apnoea (OSA) is a common chronic disease defined by the presence of repetitive episodes of upper airway collapse. OSA is a well-established risk factor for cardiovascular diseases [6, 7]. Unlike CSA, an ongoing respiratory effort is present during the apnoea events in OSA [8]; however, the overlap between CSA and OSA suggests that common mechanisms are involved [9]. Similar to OSA, CSA is associated with frequent night-time awakenings, excessive daytime sleepiness and an increased risk of adverse cardiovascular outcomes [9, 10]. Growing evidence indicates that CSA may contribute to the pathogenesis and/or prognosis of cardiovascular disease [10]. CSA is recognized as an important contributor to the progression of HF as well as HF-related morbidity and mortality [11]. Sleep fragmentation, intermittent hypoxia and sympathetic activation have been hypothesized to increase systemic blood pressure, which subsequently contributes to the development of myocardial hypertrophy and cardiac arrhythmias [12]. Moreover, the increased sympathetic activity and oxidative stress caused by the intermittent hypoxemia and arousals could cause endothelial dysfunction and predispose patients to atherosclerosis [13]. Regardless of the existing evidence on the relationship of CSA to several cardiovascular diseases, no study has assessed the impact of CSA on the severity and short-term prognosis of patients who experience ACS.

An observational study with cross-sectional and longitudinal analyses was planned to evaluate the impact of CSA on the severity and short-term prognosis of patients with ACS. The objective of the study was to compare the ejection fraction, Killip class, number of diseased vessels, peak troponin, length of hospitalization, number of complications and mortality rate of a cohort of ACS patients with CSA and a group of controls without sleep-disordered breathing (SDB).

## Methods

### Study population

**Fig 1:**
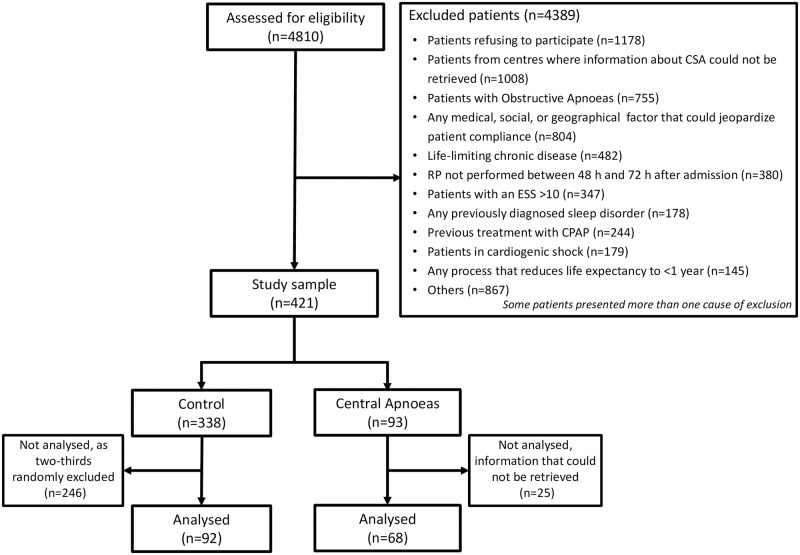
Study flowchart showing recruitment to study. CSA: central sleep apnoea; RP: cardio-respiratory polygraphy; ESS: Epworth Sleepiness Scale; CPAP: continuous positive airway pressure.

This is an ancillary study of the ISAACC study, which is a multicentre, open-label, parallel, prospective, randomized, controlled trial (registered trial NCT01335087) evaluating the effect of continuous positive airway pressure (CPAP) treatment on the incidence of new cardiovascular events in patients who have experienced both ACS and OSA [14]. The present study involved two hospitals participating in the ISAACC trial, Hospital Arnau de Vilanova, Lleida, and Hospital Clínic, Barcelona, Spain. Starting in June 2011, patients consecutively admitted for ACS to the coronary care units or cardiology hospitalization wards (male and females aged ≥18 years) were screened for the inclusion and exclusion criteria [14]. All of the patients underwent respiratory polygraphy during the first 24 to 72 h after admission. For the purpose of this analysis, patients with an apnoea-hypopnoea index (AHI) >15 events·h^-1^ and >50% central apnoeas were considered to have CSA; patients with AHI ≤15 events·h^−1^ were included as controls. OSA patients (AHI >15 events·h^-1^ with ≤50% central events) were excluded. We then compared the baseline characteristics, short-term prognosis and severity of the ACS of the patients with CSA and the controls. Fig 1 shows the detailed study flowchart.

We defined ACS as any acute presentation of coronary diseases including type 1 myocardial infarction and unstable angina [15]. The exclusion criteria for the current study included previous treatment with CPAP; psychophysical inability to complete questionnaires; the presence of any previously diagnosed sleep disorder; daytime sleepiness (Epworth Sleepiness Scale (ESS) >10); chronic disease, e.g., neoplasms, renal insufficiency (GFR <15 mL·min^−1^·1.73 m^−2^), severe chronic obstructive pulmonary disorder (a forced expiratory volume in 1 s <50%), chronic depression, and other limiting chronic diseases; a medical history that could interfere with the study objectives; any processes, whether cardiovascular or otherwise, that reduced life expectancy to <1 year; cardiogenic shock; and medical records containing greater than 50% missing data.

The ethics committee of each participating centre approved the study (approval number in the coordinator centre: 2010–852), and all patients provided written informed consent. This study was conducted according to the principles expressed in the Declaration of Helsinki.

### Data collection

The CSA diagnosis was based on the results of an overnight cardio-respiratory polygraph in accordance with the guidelines of the Spanish national consensus on the apnoea—hypopnoea syndrome [16]. All participating centres used the same polygraph model (Embletta; ResMed, Bella Vista, Australia). Oronasal flow, thoracoabdominal movements, ECG, and pulse oximetry were recorded. Apnoea was defined as an absence of airflow lasting ≥10 s. Apnoeas were considered central when no respiratory effort was detected by thoracoabdominal bands. Hypopnoea was defined as a reduction in airflow lasting ≥10 s associated with oxygen desaturation. Oxygen desaturation was defined as a decrease in arterial oxygen saturation >4%. Respiratory polygraphy studies were performed without supplemental oxygen. The AHI was defined as the number of episodes of apnoea and hypopnoea per hour of recording. The degree of self-reported sleepiness/drowsiness was analysed by the Spanish version of the ESS test [17]. An echocardiographic evaluation and Killip classification were routinely performed during each patient admission. The Killip classification focuses on the physical examination and the development of HF to predict risk. The classification considers four classes (I–IV). Class I indicates no evidence of HF, and Class IV represents cardiogenic shock. ACS severity (Killip class, ejection fraction, number of diseased vessels and peak plasma troponin) was evaluated at baseline, and short-term prognosis (length of hospitalization, complications and mortality) was evaluated at patient discharge. The cardiologists who evaluated ACS severity were blinded to the patients’ CSA or control status.

### Statistical analyses

The data for each participant were uploaded to a database. The mean ± standard deviation (SD) or frequencies (%) were computed to evaluate the differences between the CSA and control patients with respect to anthropometric measurements, clinical variables and ACS-related risk factors. The results were assessed for significance with the T test or Chi-squared test, as appropriate. The association between the CSA and the controls and the variables related to ACS severity and short-term prognosis were assessed with the T test or Chi-squared test, as appropriate. Linear and logistic regression models were used to further describe those associations. In addition, the models were adjusted for tobacco (current or former smoker versus non-smoker), age, body mass index (BMI) and hypertension. The association between the CSA patients and the controls and the length of stay in the coronary care unit (CCU) was also assessed according to CSA severity, defined as AHI ≤30 or >30 events·h^−1^. The differences in the short-term prognosis of patients with similar ACS severities were assessed by restricting the analyses to patients within the same Killip class. The data analysis was conducted using Stata 12.1 (StataCorp, College Station, TX, USA), and the threshold for significance was set at p<0.05.

### Sample size

The sample size of 68 patients with CSA and 92 controls provided a statistical power of 99.9% to detect the reported differences in the mean length of stay in the coronary care unit but only a statistical power of 34.8% to detect differences in the reported proportion of cardiovascular complications during hospitalization. The significance level was fixed at p<0.05.

## Results

A total of 68 CSA patients (AHI>15 events·h^−1^; >50% of central apnoeas) and 92 controls (AHI ≤15 events·h^−1^) were included in the analyses. Before OSA patients (n = 755) and subjects with medical records in which greater than 50% of the data were missing (n = 80) were excluded, the prevalence of CSA among ACS patients of the ISAACC cohort was 21% at Hospital Arnau de Vilanova in Lleida and 17% at Hospital Clinic in Barcelona. Anthropometric measurements, clinical variables and ACS-related risk factors for the CSA and the control patients are shown in Table 1. Among non-CSA-related variables, only the lower prevalences in diuretic (29% *vs*. 15%; p = 0.030) and oral antidiabetic (26% *vs*. 13%; p = 0.043) drug use were significant when comparing CSA patients to controls.

**Table 1:** Anthropometric, clinical and acute coronary syndrome (ACS) related variables in controls and central sleep apnoea (CSA) patients.

	Control	CSA	p-value
	AHI≤15 events/h	AHI>15 events/h	
Subjects (n)	92	68	
Age (years)	62 ±12	64 ±12	0.487
Males	77 (84%)	59 (87%)	0.591
Apnoea—hypopnoea index events/h	7 ±5	31 ±18	**<0.001**
Oxygen desaturation index >4%/h	7 ±6	19 ±18	**<0.001**
Minimum SaO_2_ (%)	85 ±6	80 ±14	**0.007**
Mean SaO_2_ (%)	93 ±3	93 ±3	0.834
Time with SaO_2_<90% (%)	9 ±19	11 ±21	0.589
Epworth Sleepiness Scale	5 ±2	4 ±2	**0.012**
Hypertensive patients	58 (63%)	42 (62%)	0.869
Body mass index (kg/m^2^)	28 ±5	28 ±4	0.851
Diabetes mellitus	27 (29%)	19 (28%)	0.846
Dyslipidaemia	52 (57%)	38 (56%)	0.936
First episode of ACS	51 (72%)	47 (69%)	0.726
Cardiomyopathy	32 (35%)	19 (28%)	0.359
Smoking			
Never	31 (34%)	17 (26%)	
Former	32 (36%)	24 (37%)	
Current	27 (30%)	24 (37%)	0.495
Diuretics	27 (29%)	10 (15%)	**0.030**
Anticoagulants	13 (14%)	6 (9%)	0.305
Antacids	32 (35%)	20 (29%)	0.473
Hypolipidemics	41 (45%)	24 (35%)	0.238
β-blockers	40 (43%)	24 (35%)	0.296
Calcium antagonists	13 (14%)	7 (10%)	0.468
Antiplatelet	22 (24%)	25 (37%)	0.078
Insulin	4 (4%)	6 (9%)	0.248
Oral antidiabetics	24 (26%)	9 (13%)	**0.043**
Bronchodilators	11 (12%)	9 (13%)	0.809

The data are presented as the mean ±SD or n (%) for quantitative or qualitative variables, respectively. SaO_2_: arterial oxygen saturation.

Table 2 shows the comparison of the variables related to ACS severity between the CSA and control patients. Although most control patients were graded as Killip I (96%), only 16% of CSA patients were graded as Killip I and the majority of CSA patients were graded Killip II (77%); these differences were highly statistically significant (p<0.001). However, no differences were identified between the groups for the peak troponin levels and the severity of HF as measured by the ejection fraction of the left ventricle.

**Table 2:** Variables related to acute coronary syndrome (ACS) severity in controls and central sleep apnoea (CSA) patients.

	Control	CSA	p-value		
	AHI≤15 events/h	AHI>15 events/h	A	B	C
Subjects (n)	92	68			
ACS category					
Unstable	19 (22%)	12 (20%)			
Non-STEMI	38 (44%)	29 (49%)			
STEMI	29 (34%)	18 (31%)	0.840	0.906^#^	0.475^#^
Killip class					
I	65 (96%)	11 (16%)			
II	3 (4%)	52 (77%)			
III	0 (0%)	5 (7%)	**<0.001**	**<0.001**^#^	**<0.001**^#^
Diseased vessels (n)					
1	37 (56%)	29 (47%)			
2	15 (23%)	23 (37%)			
≥3	14 (21%)	10 (16%)	0.202	0.760^#^	0.765^#^
Stents implanted (n)	1.13 ±0.64	1.34 ±1.17	0.258	0.258	0.255
Ejection fraction (%)	55.9 ±12	57.4 ±13	0.488	0.488	0.262
Peak troponin I (ng/ml)	29.6 ±61	20.1 ±41	0.297	0.297	0.481
Altered peak troponin I (≥0.04 ng/ml)	83 (90%)	60 (88%)	0.688	0.688	0.469

The data are presented as the mean ±SD or n (%) for quantitative or qualitative variables, respectively, unless otherwise stated. AHI: apnoea—hypopnoea index; STEMI: ST-elevation myocardial infarction. p-values evaluated the differences between groups using a t test or Chi-squared test as appropriate (A); linear and logistic regression models (B); and regression models adjusted for age, sex, body mass index, tobacco use (current or former smoker versus non-smoker) and hypertension (C).

^#^: ordinal integer values considered in the linear model analyses to evaluate the differences in the trend for the ACS category, Killip classification and number of diseased vessels.

**Fig 2:**
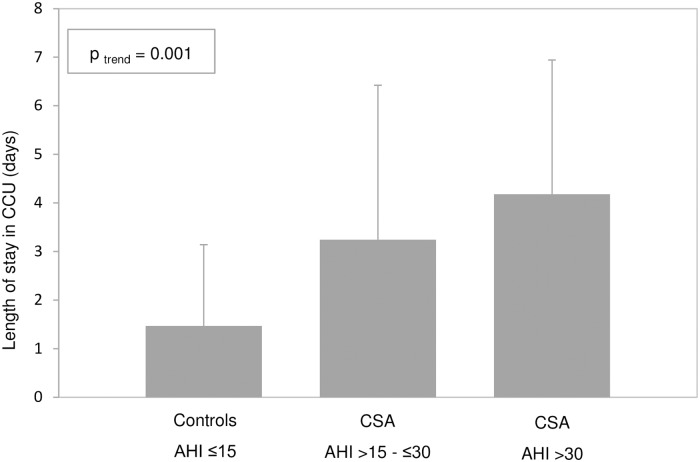
Mean length of stay in the coronary care unit according to central sleep apnoea severity. CCU: coronary care unit; CSA: central sleep apnoea; AHI: apnoea—hypopnoea index. P-values correspond to a model using CSA severity categories as a continuous variable, adjusted by age, sex, body mass index, tobacco (current or former smoker versus non-smoker) and hypertension.

The differences between the CSA and control patients in the variables related to the short-term prognosis following ACS are presented in Table 3. The length of stay in the CCU was significantly longer for CSA patients (3.7 ±2.9days compared with 1.5 ±1.7 days for controls, p<0.001). When this relationship was analysed according to CSA severity, defined as AHI ≤30 or >30 events·h^−1^, a significant trend towards longer CCU stays according to CSA severity was revealed (Fig 2). In this sense, it is reassuring that CSA patients were more prone to cardiovascular complications during hospitalization than controls (24% vs. 10%, p = 0.058), although this difference did not reach statistical significance due to low statistical power. The main cardiovascular complications in our study were arrhythmias, heart failure, pulmonary oedema, hypertensive crisis, re-infarction, and cardiac arrest. Notably, only 2 CSA patients died during their hospital stay.

**Table 3:** Variables related to the short-term prognosis of acute coronary syndrome (ACS) in controls and central sleep apnoea (CSA) patients.

	Control	CSA	p-value		
	AHI≤15 events/h	AHI>15 events/h	A	B	C
Subjects (n)	92	68			
Length of stay in the CCU (days)	1.5 ±1.7	3.7 ±2.9	**<0.001**	**<0.001**	**<0.001**
Length of hospitalization (days)	7.3 ±5.5	7.3 ±5.6	0.947	0.947	0.489
CV complications during hospitalization	6 (10%)	16 (24%)	0.052	0.058	0.058
Mortality during hospitalization	0 (0%)	2 (3%)	0.181	-	-

The data are presented as the mean ±SD or n (%) for quantitative or qualitative variables, respectively, unless otherwise stated. AHI: apnoea—hypopnoea index; CCU, coronary care unit; CV: cardiovascular. p-values evaluated differences between the groups using a t test or Chi-squared test as appropriate (A); linear and logistic regression models (B); and regression models adjusted for age, sex, body mass index, tobacco use (current or former smoker versus non-smoker) and hypertension (C).

The comparison between Killip I CSA patients (n = 11) and Killip I controls (n = 65) revealed a significant difference in the length of stay in the CCU (1.4 ±1.5 days for controls, 5 ±4.4 days for CSA patients; p<0.001).

## Discussion

In this observational study of patients who experienced ACS, we assessed the relationship between CSA and the severity and short-term prognosis of ACS. In patients with ACS, the diagnosis of CSA was related to a higher Killip class and lower use of diuretics and oral antidiabetics. CSA was associated with a longer length of stay in the coronary care unit.

The prevalence of CSA in patients with cardiovascular diseases has been reported to be high. Hetzenecker et al. reported a CSA prevalence of 21.8% in their study of 55 consecutive patients with acute myocardial infarction (AMI) who underwent subsequent percutaneous coronary intervention [18]. On the other hand, prevalences ranging from 33 to 40% have been described in HF patients [4,19]. In our cohort, the CSA prevalence was 20.1%, which is comparable with Hetzenecker’s results and supports the hypothesis that ACS patients have a lower prevalence of CSA than HF patients. Hetzenecker’s study of AMI patients revealed that patients with SDB were older, more likely to be men, had higher BMIs, lower ejection fractions and a higher Killip class than non-SDB patients. However, no specific results for the diagnosis of CSA were reported [18]. Previous studies in HF patients have reported that CSA patients were older, more likely to be men and had a higher BMI than controls [19–21]. In our study, no differences were identified between the CSA patients and the controls regarding age, gender or known ACS risk factors, such as BMI, smoking or hypertension. Moreover, no differences regarding the severity of HF (measured by the ejection fraction of the left ventricle) were noted. Although CSA patients did not differ from controls in regard to key risk factors for ACS, we identified CSA as a negative prognostic factor in myocardial ischemia and identified differences in ACS severity (Killip grade) and short-term prognosis (days in the CCU and CV complications during hospitalization) in CSA patients compared with controls. CSA has adverse prognostic implications in HF patients [4]. Similarly, a study that examined cerebral ischemic strokes in patients with and without CSA revealed that CSA is a negative prognostic factor in acute stroke and is related to stroke severity, topography and systolic dysfunction [22]. Therefore, our findings reinforce the close association between CSA and cardiovascular diseases and implicate ACS as a cardiovascular pathology related to CSA.

Patients with HF and CSA tend to have an exaggerated respiratory response to carbon dioxide that is associated with excess sympathetic nervous activity. In such patients, elevated telediastolic ventricular pressures cause pulmonary congestion, leading to central activation of breathing by means of vagal activation and diminishment of the PaCO_2_ reserve. Subsequent hyperventilation causes PaCO_2_ to drop below the apnoea threshold and results in the occurrence of an apnoea episode. Finally, cessation of breathing leads to accumulation of CO_2_, which is the chemical stimulus for resumption of ventilation [23–26]. This malfunction in the homeostatic feedback loop is exacerbated by the prolonged circulation time between the alveoli and brainstem [26]. However, in the absence of HF, it is unlikely that this mechanism could explain the relationship between ACS and CSA. A small study by Lanfranchi concluded that CSA severity might not be related to the severity of hemodynamic impairment. However, severe CSA was associated with impaired cardiac autonomic control and with increased cardiac arrhythmias [27]. It is noteworthy that we did not identify differences in the ejection fraction between CSA and non-SDB patients. In contrast, previous studies have demonstrated that OSA is an independent risk factor for myocardial infarction and other coronary events and is related to a worse CV prognosis [13, 28–30]. Given the physiopathological mechanisms shared between OSA and CSA, it is possible that the chronic intermittent hypoxia and sleep fragmentation caused by SDB are the main links between CSA and ACS. However, it must be noted that the reported differences between controls and CSA patients differ from the differences observed between controls and OSA patients in the previously published results from the ISAACC study [28]. Systemic inflammation induced by chronic intermittent hypoxia could increase atherogenesis in OSA [29], thus serving as a plausible mechanism for the increased cardiovascular risk due to CSA. Similarly, increased sympathetic tone, oxidative stress, hypercoagulability and cardiac hyper-excitability caused by intermittent episodes of hypoxemia, reoxygenation and brain arousals could also be key elements in the relationship between ACS and SDB [31]. Finally, it is noteworthy that SDB is related to infarct expansion, impaired healing of myocardial tissue and coronary artery plaque burden [32–34].

Our results highlight the relevance of CSA in patients who have suffered from an ACS episode. CSA could be suspected in patients of Killip grade II or higher. Regardless of their Killip class, in our study, the CSA patients tended to have longer stays in the CCU and exhibited a greater risk of cardiovascular complications (including death) compared with non-SDB patients. Therefore, the identification of CSA with a sleep test could prove useful for the management of ACS patients. If these results are confirmed, future studies should evaluate whether successfully treating CSA could improve the prognosis and severity of ACS in these patients.

The current study has several strengths, including the novelty of studying the relationship between CSA and ACS and the evaluation of a broad range of variables, which includes variables related to ACS severity and short-term prognosis. In addition, the following limitations should be acknowledged. (i) The cross-sectional design precludes any interpretation about the directionality of the association between CSA and ACS. Interestingly, Kasai et al. suggested that the relationship could even be bi-directional [23]. (ii) The limited number of subjects precluded the modelling of mortality, and the low heterogeneity in the Killip class limited stratified analyses by ACS severity. (iii) Sleepy subjects (ESS>10) were excluded from the current analyses due to the ethical restrictions of the ISAACC study, which has a non-CPAP arm. However, few patients were excluded based on this criterion, and this exclusion should not affect our conclusions as subjects reporting day-time sleepiness are generally prone to visit to sleep units.

## Conclusions

In the present study, patients with CSA exhibit increased ACS severity as indicated by their Killip classification. Moreover, these patients exhibited a worse prognosis with a longer length of stay in coronary care units and more complications during hospitalization, regardless of their Killip class. Overall, our results highlight the relevance of CSA in patients who experience ACS and suggest that using a sleep test to diagnose CSA may be a useful strategy to improve the management of certain ACS patients.
